# FSTL3 is a biomarker of poor prognosis and associated with immunotherapy resistance in ovarian cancer

**DOI:** 10.1186/s13046-025-03425-4

**Published:** 2025-09-30

**Authors:** Maeva Chauvin, Estelle Tromelin, Julien Roche-Prellezo, Hyshem H. Lancia, Marie-Charlotte Meinsohn, Caroline Coletti, Ngoc Minh Phuong Nguyen, Virginie Lafont, Henri-Alexandre Michaud, Ranjan Mishra, Nathalie Bonnefoy, Laurent Gros, David Pépin

**Affiliations:** 1 Department of Surgery, Pediatric Surgical Research Laboratories, Massachusetts General Hospital, Harvard Medical School, Boston, MA 02114 USA; 2 Institut de Recherche en Cancérologie de Montpellier, INSERM U1194, Université de Montpellier, Institut régional du Cancer de Montpellier, Montpellier, France; 3 Spatial Imaging Mass Cytometry and Transcriptomics Platform, Institute de Recherche en Cancérologie de Montpellier, INSERM U1194 - UM – ICM, Montpellier, France; 4 Department of Surgery, Center for Transplantation Sciences, Massachusetts General Hospital, Harvard Medical School, Boston, MA USA; 5 Whitehead Institute for Biomedical Research, Cambridge, MA USA; 6 Department of Obstetrics and Gynecology, University of Rennes, Rennes, France

## Abstract

High-grade serous ovarian carcinoma (HGSOC) is associated with high mortality rates due to late-stage diagnosis and limited treatment options. We investigated the role of FSTL3 in ovarian cancer progression both as a prognostic biomarker and as a potential therapeutic target.

We measured levels of follistatin (FST) and follistatin-like 3 (FSTL3) in 96 ovarian cancer patient ascites samples and found that FSTL3 overexpression was more predominant than FST and associated with poorer survival outcomes. Mice implanted with an HGSOC syngeneic cell line bearing common alterations in ovarian cancer (KRAS^G12 V^, P53^R172H^, CCNE1^oe^, AKT2^oe^) had increasing levels of FST and FSTL3 in serum during tumor growth. Further alteration of this model to generate a knockout of FST (KPCA.FSTKO) and an overexpression of human FSTL3 (KPCA.FSTKO_hFSTL3) revealed that FSTL3 expression was associated with a more fibrotic tumor microenvironment, correlating with an increased abundance of cancer-associated myofibroblasts (myCAFs), and cancer cells with a more mesenchymal phenotype. Tumors overexpressing FSTL3 also had significantly less immunocyte infiltration, reduced intratumoral T-cell abundance, and increased CD8+ T cell exhaustion. FSTL3 overexpression completely abrogated tumor response to PPC treatment (Prexasertib combined with PD-1 and CTLA-4 blockade) compared to controls, suggesting that FSTL3 may be involved in immunotherapy resistance.

In conclusion, this study suggests a role for FSTL3 as a prognostic marker and as therapeutic target in HGSOC, where it may play a role in promoting a mesenchymal tumor phenotype, maintaining an immunosuppressive tumor microenvironment, and driving immunotherapy resistance.

**Graphical Abstract:**

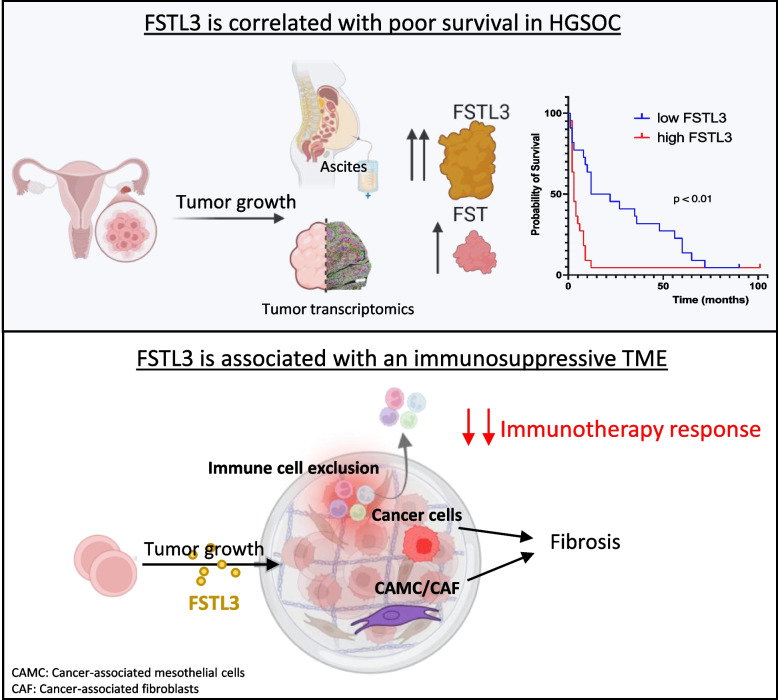

**Supplementary Information:**

The online version contains supplementary material available at 10.1186/s13046-025-03425-4.

## Highlights

• High FSTL3 levels in patient ascites fluid is associated with poor outcomes in ovarian cancer.

• Serum levels of FSTL3 reflect tumor burden and therapy response in mice.

• Overexpression of *Fstl3* in a syngeneic mouse ovarian cancer model promotes a fibrotic tumor microenvironment and immunocyte exclusion.

• Overexpression of *Fstl3* in tumors induces resistance to Chk1 and immune checkpoint inhibitor combination therapy.

**Supplementary Information:**

The online version contains supplementary material available at 10.1186/s13046-025-03425-4.

## Introduction

Ovarian Cancer is the 8th most common cancer among women worldwide [1, 2], with over 324,000 new cases and approximately 207,000 deaths reported in the world in 2022, making it the 5th leading cause of cancer-related mortality in women [1, 2]. Projections indicate that by 2050, the worldwide incidence of ovarian cancer will increase by over 55%, and the number of deaths due to ovarian cancer is set to rise by almost 70%, to a total of 350,956 per year [1, 2]. The high mortality rate is largely attributed to late-stage diagnosis, as the disease often presents with nonspecific symptoms. Ovarian carcinoma accounts for over 90% of all ovarian cancer cases, with high-grade serous ovarian carcinoma (HGSOC) being the most prevalent and aggressive subtype [3, 4]. The standard treatment for ovarian cancer remains debulking surgery combined with platinum-based chemotherapy. Despite optimal treatment, prognosis remains poor, with 70–80% of patients experiencing relapse, and a 5-year survival rate of only 17% for those diagnosed at advanced stages, primarily due to resistance to platinum-based therapies [5, 6].

Platinum-resistant recurrent HGSOC, particularly in BRCA wild-type cases (which represent approximately 75% of all HGSOC cases) poses a significant therapeutic challenge due to limited treatment options [7–9]. This highlights the urgent need to develop novel therapeutic agents. Despite the high prevalence of CD4+ and CD8+ T cells, immune checkpoint blockade therapies, designed to enhance anti-tumor immune responses, have demonstrated limited efficacy in ovarian cancer [10–12]. This may be attributed to the tumor microenvironment, which is adept at suppressing immune responses through immunosuppressive cells and cytokines, thereby driving immunotherapy resistance [13–15].

Recent advancements in preclinical models have provided valuable insights into such treatment resistance. The KPCA syngeneic mouse model of CCNE1-amplified HGSOC bears KrasG12 V and Trp53R172H mutations and overexpresses Ccne1 and Akt2 [16]. We have previously observed significant variability in therapeutic responses among genetically identical cell line clones of KPCA treated with a combination of immunotherapy (anti-PD-L1/CTLA-4) and targeted therapies (Prexasertib, a CHK1 inhibitor) collectively referred to as PPC, ranging from clones with complete sensitivity (KPCA.B) to complete resistance (KPCA.C). When comparing the transcriptomes of these clones we identified follistatin (FST) as a potential immunomodulatory factor overexpressed in tumors through epigenetic means and associated with resistance to PPC treatment [16]. To further investigate the mechanisms underlying this resistance, the follistatin gene was deleted in KPCA.C resistant clone (KPCA.FSTKO), which restored sensitivity to PPC; likewise, overexpression of FST in the sensitive KPCA.B clone (KPCA.B-FST^OE^) rendered it resistant to PPC treatment [16]. We hypothesized that follistatin overexpression inhibits immune responses, and thus promotes immunotherapy resistance, through the neutralization of TGF-β superfamily ligands, such as Activin A, which play a critical role in regulating multiple cells of the tumor microenvironment (TME), including cancer cells and immunocytes [17, 18]. Elevated follistatin expression in tumors was associated with worse overall survival in CCNE1-amplified HGSOC, and its presence in blood may serve as an early diagnostic marker for ovarian cancer [19].

Building on these findings, we sought to investigate the role of follistatin-like-3 (FSTL3), a close paralog of FST with overlapping TGF-β superfamily ligand affinity [20]. A recent study in colorectal cancer suggested that FSTL3 may contribute to immune evasion and showed that FSTL3 overexpression in malignant cells was linked to poor clinical survival outcomes with anti-PD1 therapy [21]. In immunocompetent tumor models, FSTL3 knockout led to an increased proportion of CD8+ T cells and a reduction in regulatory T cells and exhausted T cells, improving the efficacy of anti-PD1 therapy [21]. These data suggest that FSTL3 could serve as both a biomarker for immunotherapeutic efficacy and a novel therapeutic target to sensitize tumors to anti-PD1 treatment [21].

This study aimed to understand how FSTL3 contributes to ovarian tumor development and aggressiveness, and if it regulates the tumor immune microenvironment. We explored the role of FSTL3 independently of FST by overexpressing human FSTL3 in the KPCA.FSTKO cell line, which we will refer to as KPCA.FSTKO_hFSTL3. Herein, we found that overexpression of FSTL3 changed the tumor microenvironment, leading to increased fibrosis and immune exclusion, and caused resistance to combination immunotherapy (PPC treatment).

## Results

### Clinical Investigation of FST and FSTL3 in Ovarian Cancer: Insights from Patient Samples 

**Fig. 1:**
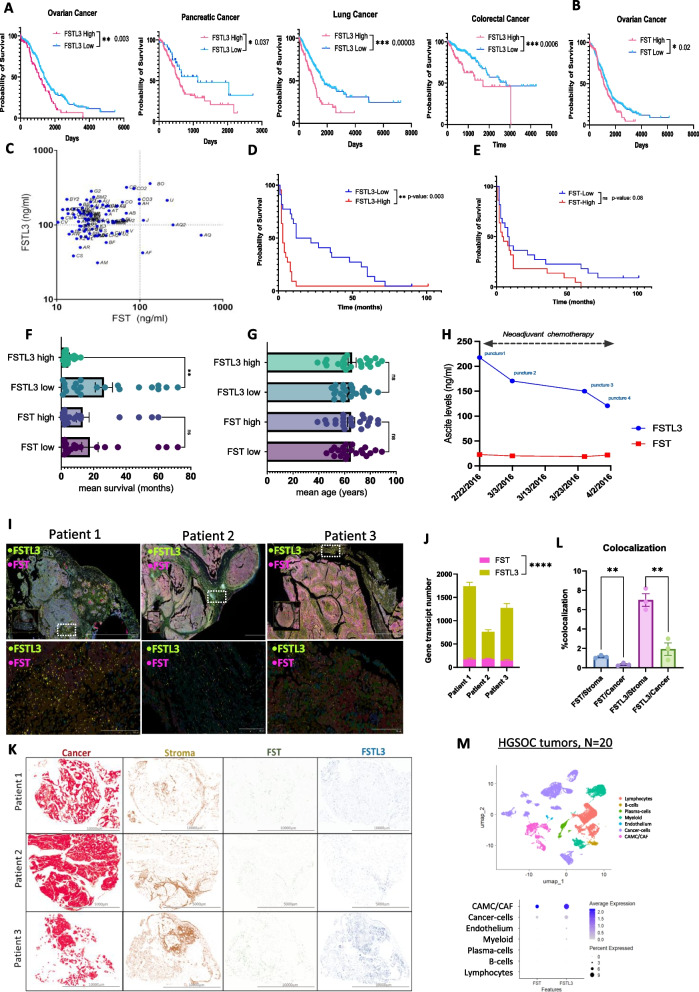
FSTL3 is overexpressed in ascites and ovarian tumor sections and is associated with poor survival. **A** Kaplan-Meier survival plots comparing patient populations with low and high FSTL3 and **B** FST transcriptomic expression according to TCGA datasets. **C** Concentration of FST and FSTL3 in 96 ascites samples from patients with various ovarian cancer subtypes. **D**,**E** Kaplan-Meier survival curves comparing low and high FST and FSTL3 protein levels in 44 patients with HGSOC, BRCA1/2 mutation-negative (groups defined by median expression levels). **F** Mean survival (in months) comparing patients with low and high FST and FSTL3 levels from panels **D**,**E**. **G** Mean age distribution across groups from panel **D**,**E**. **H** FST and FSTL3 protein levels measured in ascites during neoadjuvant chemotherapy in a 44-year-old patient with HGSC. **I** Spatial transcriptomics was performed on HGSOC tissue samples from three patients using the Xenium platform (10X genomics). Representative sections showing the spatial distribution of expression of FST and FSTL3 transcripts within the tissue microenvironment. **J** Number of transcripts of FST and FSTL3 across 12 images covering the entire tumor area per patient (*N*=3). **K** Spatial expression of markers of cancer cells (Epcam+, red color), stroma (Upk3b+, Acta2+, Pdgfr+, brown color), and FST (green color) and FSTL3 (blue color) in whole tumor sections. **L** Quantification of colocalization by representing the percentage of area overlap between FST or FSTL3 and either stromal or epithelial markers. **M** UMAP and Dot plot showing the expression of FST and FSTL3 in cell populations from 20 treatment-naïve human ovarian tumors (HGSOC) (*N*=20)

Analysis of The Cancer Genome Atlas (TCGA) datasets revealed that FSTL3 was associated with poor survival outcomes in multiple cancers, including ovarian, colorectal, pancreatic and lung cancers (Fig. 1A); in comparison, FST had a weaker correlation of poor prognosis for ovarian cancer (Fig. 1B). To evaluate the relative expression of FST and FSTL3 in ovarian cancer, we measured the concentrations of these proteins by ELISA in 96 ascites samples from 77 patients with various peritoneal tumors of ovarian cancer and other origins, both chemo-naïve and treated (Supplementary Material 2, Table S1). We found that FST expression in ascites (mean: 42.5 ng/ml) was substantially elevated, ranging from 10- to 139-fold higher compared to reported values in normal peritoneal fluid (1.8 ng/mL) and blood serum (3.5 +/- 0.2 ng/ml) [22], or ascites of endometrioma patients (9.8ng/ml) [19, 23]. Furthermore, FSTL3 levels in ascites (mean: 130.9ng/ml) were significantly higher than those of FST (*p*<0.0001), with a mean fold increase of 5.14 (Fig. 1C) (Supplementary Material 2, Table S1). Although normal FSTL3 concentrations in peritoneal fluid are not well documented, these levels ranged from 10- to 100-fold higher than typical serum concentrations reported in the literature (mean: 3.4 ng/ml) [24].

#### High FSTL3 levels in ascites are associated with poor survival outcomes in HGSOC

Next, we sought to examine the correlation between FST and FSTL3 levels in ascites and overall survival. To reduce variability and improve clinical relevance, we focused our analysis on a more homogenous group of 44 patients with high-grade serous ovarian cancer and BRCA wild-type genotype. We found that elevated FSTL3 levels were significantly associated with reduced survival rates (*p*=0.0035) (Fig. 1D). Notably, when dividing patients into two groups based on the median FSTL3 expression (median = 109.8 ng/mL, IQR [75.65–143.5]), the group with higher FSTL3 levels had a significantly shorter mean survival of 4.38 ± 0.67 months, compared to 26.33 ± 5.33 months in the group with lower FSTL3 levels (*p<*0.01) (Fig. 1F).

Although the correlation between FST levels and survival was less pronounced (*p* = 0.088) (Fig. 1E), we observed a mean survival of 17.7 ± 5.06 months in the group with lower FST expression (median = 26.00 ng/mL, IQR [21.35–38.21]), compared to 13.23 ± 3.96 months in the group with higher FST expression (Fig. 1F). However, this difference was not statistically significant. These findings were consistent with previous TGCA analysis (Fig. 1A/B). Importantly, no significant correlation was observed between patient age and the expression levels of either FST (mean: 42.5ng/ml) or FSTL3 (mean: 130.9ng/ml) (Fig. 1G, Supplementary Material 2, Table S1). Finally, when FST and FSTL3 levels were measured in ascites samples from a 44-year-old patient with HGSOC during her course of neoadjuvant chemotherapy (Carboplatin-Taxol), a reduction in FSTL3 but not FST levels was observed over time (Fig. 1H), suggesting FSTL3 concentration in ascites fluid may better reflect response to therapy.

#### FST and FSTL3 are predominantly secreted by tumor stromal cells

Next, we aimed to investigate the expression levels of FST and FSTL3 in human ovarian tumor tissues. To achieve this, we employed spatial transcriptomic analysis using the Xenium platform, a cutting-edge technology that enables high-resolution mapping of gene expression on tissue slides. Tissue sections were obtained from three treatment-naive high-grade serous ovarian cancer (HGSOC) tumors. We quantified gene transcripts using the Xenium analysis software, with transcript counts averaged across 12 non-overlapping regions of interest per patient to ensure a comprehensive representation of all tumor areas and capture sample heterogeneity within the tumor microenvironment (Fig. 1I,J). Across the three patients analyzed, the mean transcript count for FSTL3 was 1094.64, which was significantly higher than FST with a mean transcript count of 161.75 (Fig. 1J). These findings highlight the predominance of FSTL3 expression in tumor tissues, which parallels our findings in ascitic fluid.

We next investigated the spatial pattern of expression of FST/FSTL3 to understand the relative contributions of cancer cells and stromal cells to their secretion in the TME. The mapping of key tumor microenvironmental markers revealed a preferential localization of FST and FSTL3 within the stromal compartment (Fig. 1K/L). Cancer cells, identified by Epcam expression (red staining), showed minimal expression of both factors, with colocalization below 2%. In contrast, stromal regions positive for markers such as Upk3b (CAMC), Acta2, and Pdgfr (CAF) (brown staining) exhibited high FST (green) and FSTL3 (blue) signals, with colocalization reaching up to 4% and 8% respectively, suggesting that these proteins are primarily secreted by stromal cells (Fig. 1K). Quantification of colocalization was performed using the Coloc2 plugin in ImageJ and corresponds to the percentage of area overlap between FST or FSTL3 and either stromal or Epcam markers. To further validate this observation, we analyzed the expression of FST and FSTL3 at the single-cell level in a cohort of 20 treatment-naïve high-grade serous ovarian carcinoma (HGSOC) tumors (datasets GSE235931 and GSE241221). Dot plot analysis demonstrated that FST and FSTL3 were expressed in stromal cell populations of cancer-associated mesothelial cells (CAMC) and cancer-associated fibroblasts (CAF), at a higher level than in cancer cells (Fig. 1L). These findings suggest that stromal cells are the primary source of FST and FSTL3 secretion in the ovarian tumor microenvironment.

#### Serum levels of FST and FSTL3 increase during tumor growth in mice

**Fig. 2:**
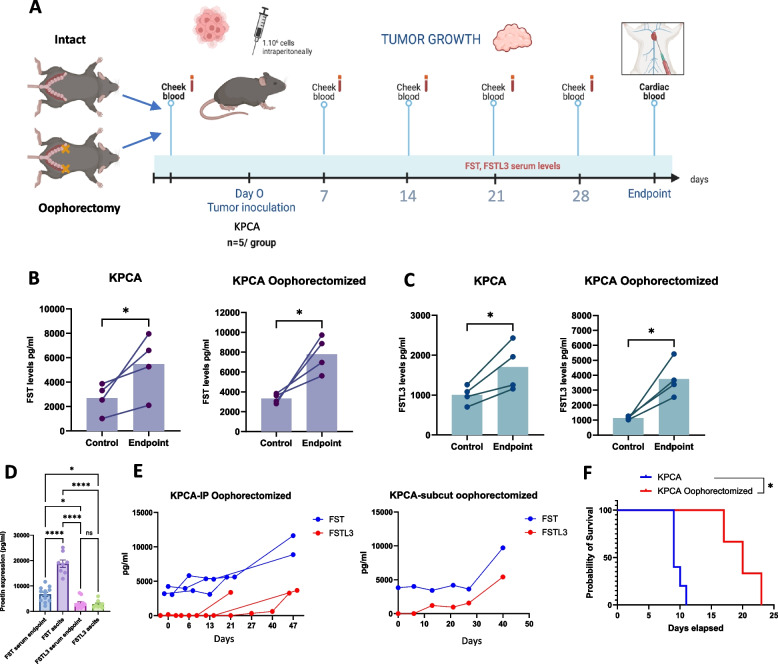
FST and FSTL3 serum levels correlate with tumor growth. **A** In vivo experimental design outlining the timepoints of blood collection during tumor growth following implantation with KPCA.C cells in mice after oophorectomy, or in intact mice. **B**,**C** Serum levels of FST and FSTL3 in intact and oophorectomized mice before grafting and at endpoint. **D** ELISA measurements of FST and FSTL3 expression in serum at endpoint and in ascites. **E** Serum levels of FST and FSTL3 during tumor progression in mice injected intraperitoneally (IP) with KPCA cells (*N* = 3) or bearing subcutaneous KPCA grafts (*N* = 1), all in oophorectomized mice. **F** Kaplan–Meier survival curves comparing KPCA graft models in intact versus oophorectomized mice

To determine if FST and FSTL3 serum levels correlate with tumor growth and can serve as biomarkers of tumor burden, we monitored their levels in a syngeneic mouse model of CCNE1-amplified high-grade serous carcinoma (KPCA.C cell line clone), which we have previously reported secretes FST (~50pg/ml in KPCA supernatant) [25]. To better mimic the post-menopausal status of most HGSOC patient, and control for the high ovarian secretion FST [26], we included oophorectomized mice in our measurements of serum levels of FST and FSTL3 by ELISA (Fig. 2). The KPCA cells were injected 3 weeks after the oophorectomy was performed. Blood samples were collected through submandibular vein puncture (cheek blood) and via cardiac puncture at endpoint.

We observed a significant increase in FST and FSTL3 serum levels by ELISA at endpoint compared to pre-graft levels in the KPCA tumor bearing mice (Fig. 2A, B) (*N*=4 mice per group). Similarly, in oophorectomized mice, FST and FSTL3 levels rose significantly during tumor growth. Surprisingly, oophorectomy in mice did not significantly alter the FST concentration between the pre-graft and endpoint measurements, suggesting there may be other confounding sources of FST. In addition, we compared the levels of both proteins in serum and ascites collected at endpoint. Surprisingly, FST concentrations were higher in ascites than in serum, whereas FSTL3 levels were similar in both fluids (Fig. 2D). This result suggests that FST may have limited diffusion into the bloodstream due to its binding to components in the ascites or faster degradation in the serum.

Longitudinal monitoring of FST and FSTL3 in serum, regularly tracked during tumor growth, revealed a similar upward trend for both proteins over time (Fig. 2E). Interestingly, ovariectomized mice grafted with KPCA cancer cells exhibited a significant prolongation of survival (*p*<0.05) compared to intact mice, suggesting an influence of ovarian hormones in promoting tumor growth (Fig. 2F).

#### FSTL3 Overexpression induces resistance to PPC treatment

**Fig. 3:**
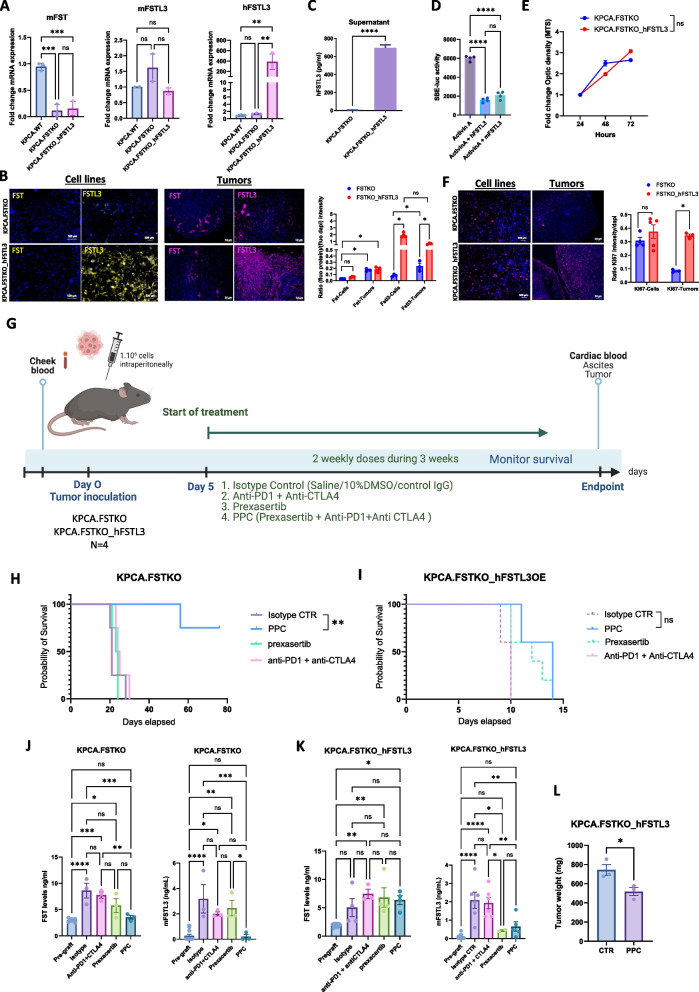
FSTL3 overexpression causes immunotherapy resistance. **A** qPCR analysis of mouse Fst (mFST), mouse Fstl3 (mFSTL3), and human FSTL3 (hFSTL3) expression in KPCA wild-type (WT), KPCA.FSTKO, and KPCA.FSTKO-hFSTL3 models. **B** Representative images and quantification of FST and FSTL3 protein expression in cell lines and tumors from KPCA.FSTKO and KPCA.FSTKO-hFSTL3 models. Fluorescence intensity was quantified using ImageJ software and normalized to DAPI signal. **C** ELISA measurement of hFSTL3 levels in the supernatant of KPCA.FSTKO and KPCA.FSTKO-hFSTL3 cells. **D** SBE-luciferase reporter cells, which express a Smad-binding element (SBE)-driven luciferase construct, were used to measure Activin A signaling activity. Cells were stimulated with recombinant mouse Activin A (1ng/mL) in the presence or absence of recombinant human FSTL3 or murine FSTL3 (2ng/ml). Luciferase activity was measured 16 hours post-stimulation. **E** MTS proliferation assay comparing KPCA.FSTKO and KPCA.FSTKO-hFSTL3 models over time (24 h, 48 h, and 72 h). **F** Representative images and quantification of FST, FSTL3, and Ki67 protein expression in cell lines and tumors from KPCA.FSTKO and KPCA.FSTKO-hFSTL3 models. Fluorescence intensity was quantified using ImageJ software and normalized to DAPI signal. **G** In vivo experimental design outlining the treatment groups and methodology used to evaluate PPC immunotherapy response. **H** Validation of hFSTL3 overexpression by ELISA comparing the supernatant of media conditioned by the KPCA.FSTKO parental and chosen KPCA.FSTKO_hFSTL3 clone. **I** Kaplan-Meier survival curves comparing the survival outcomes of KPCA.FSTKO and **J**) KPCA.FSTKO_hFSTL3 models following treatment with control (IgG isotype + vehicle), Prexasertib, anti-PD1 + anti-CTLA4, or the PPC triple combination (Prexasertib + anti-PD1 + anti-CTLA4) by intra-peritoneal injections 2 times/week (*N*=4/groups) **K**) Comparison of FST and FSTL3 serum levels pre-graft and at endpoint across all treatment groups in KPCA.FSTKO and **F**) KPCA.FSTKO_hFSTL3 grafted mice. L) Tumor weight at endpoint (day 10) in the KPCA.FSTKO_hFSTL3 model treated or not with PPC

To better understand the consequence of FSTL3 overexpression on ovarian tumor growth and response to therapy (Fig 3E), we used the parental cell line KPCA.FSTKO to generate a cell line overexpressing human FSTL3 (KPCA.FSTKO_hFSTL3). Gene expression analysis by qPCR (Fig. 3A) confirmed the complete loss of *Fst* expression in both KPCA.FSTKO and KPCA.FSTKO-hFSTL3 cells, while endogenous murine *Fstl3* expression remained low and unchanged in both cell lines. Human FSTL3 expression was detected exclusively in KPCA.FSTKO-hFSTL3 cells (Fig. 3A).

Protein expression was further assessed by immunofluorescence in both cell lines and in tumors. Quantification in cell lines confirmed the knockout of FST and low expression of FSTL3, while in tumors, FST and FSTL3 expression were significantly higher in KPCA.FSTKO tumors, indicating stromal-derived expression within the tumor microenvironment (Fig. 3B). Secretion of human FSTL3 protein into the culture supernatant was also confirmed by ELISA (Fig. 3C).

Next, we assessed whether human FSTL3 could inhibit mouse Activin A ligand, and found that it had a similar inhibitory efficacy as murine FSTL3 (Fig. 3D). We also evaluated cell proliferation using MTS assays, which showed no significant difference in proliferation between KPCA.FSTKO and KPCA.FSTKO-hFSTL3 cells (Fig. 3E), a result further supported by immunofluorescence analysis of Ki67 expression in these cell lines (Fig. 3F). In contrast, staining of tumor sections revealed higher Ki67 expression in KPCA.FSTKO-hFSTL3-derived tumors than in KPCA.FSTKO controls (Fig. 3F). These results suggest non-cell-autonomous proliferative effects of FSTL3 in the TME.

Next we sought to understand if FSTL3 overexpression could phenocopy the effect of FST on immunotherapy resistance in the KPCA model. The KPCA.C syngeneic clone is inherently resistant to PPC treatment, which includes a combination of anti-PD1 (50ug), anti-CTLA4 (50ug), and the CHK1 inhibitor Prexasertib (10 mg/kg), treated with two IP injections per week [16]. Previous findings indicated that the KPCA.FSTKO model, achieved through genetic ablation of *Fst* in KPCA.C, restored the sensitivity to PPC treatment [16]. We hypothesized that FSTL3, being a close homolog of FST, could also induce resistance to PPC. As previously reported, we found that individual therapies such as Prexasertib, or a combination of immune checkpoint blockade alone (anti-PD1 plus anti-CTLA4) were not sufficient to prolong the survival of mice implanted with KPCA.FSTKO cells. However, the combination of all three therapies (anti-PD1 plus anti-CTLA4 plus Prexasertib) or “PPC” was synergistic and significantly improved survival with 80% of the mice tumor-free at the end of the study (*p*<0.01) (Fig. 3G).

In contrast, overexpression of FSTL3 caused resistance to PPC in our KPCA.FSTKO_hFSTL3 model (Fig. 3H), indicating that overexpression of FSTL3 phenocopies FST-mediated resistance to PPC, which failed to significantly improve overall survival, albeit leading to slightly smaller tumors at endpoint (F ig. 3I).

#### PPC therapy restores physiological FST and FSTL3 levels

We next sought to determine if the therapeutic response to PPC, and the corresponding decrease in tumor burden would be reflected in FST or FSTL3 levels in serum at endpoint. We observed significant increases in FST and FSTL3 levels when comparing pre-graft levels (FST 2.9 ng/ml, FSTL3 0.15 ng/ml) to endpoint levels following grafting with KPCA.FSTKO cells and treatment with either isotype + vehicle control (FST 8.6 ng/ml, FSTL3 3.1 ng/ml), anti-PD1 + anti-CTLA4 (FST 7.7 ng/ml; FSTL3 2 ng/ml), or Prexasertib (FST 5.7 ng/ml; FSTL3 2.45 ng/ml) alone, reflecting the muted effect of these treatments on survival. In contrast, PPC treatment significantly reduced FST and FSTL3 levels compared to isotype control and restored both FST (~3.5ng/ml) and FSTL3 (~0.19ng/ml) to physiological levels (Fig. 3J).

In the mice grafted with KPCA.FSTKO_hFSTL3 cells, none of the treatments caused significant changes in the FST levels, suggesting overexpression of hFSTL3 induced resistance to PPC treatment in these tumors (Fig. 3K). Interestingly, when measuring endogenous mouse FSTL3 levels with a species-specific ELISA, we observed a significant reduction in concentration with prexacertib and PPC compared to isotype control (Fig. 3K). This decline in murine FSTL3 may be due to a modest effect of PPC treatment on tumor burden reduction (fold change: 1.31), as observed at endpoint (*p* < 0.05), which failed to change survival (Fig. 3L).

#### FSTL3 promotes tumor growth and is associated with a more fibrotic microenvironment

**Fig. 4:**
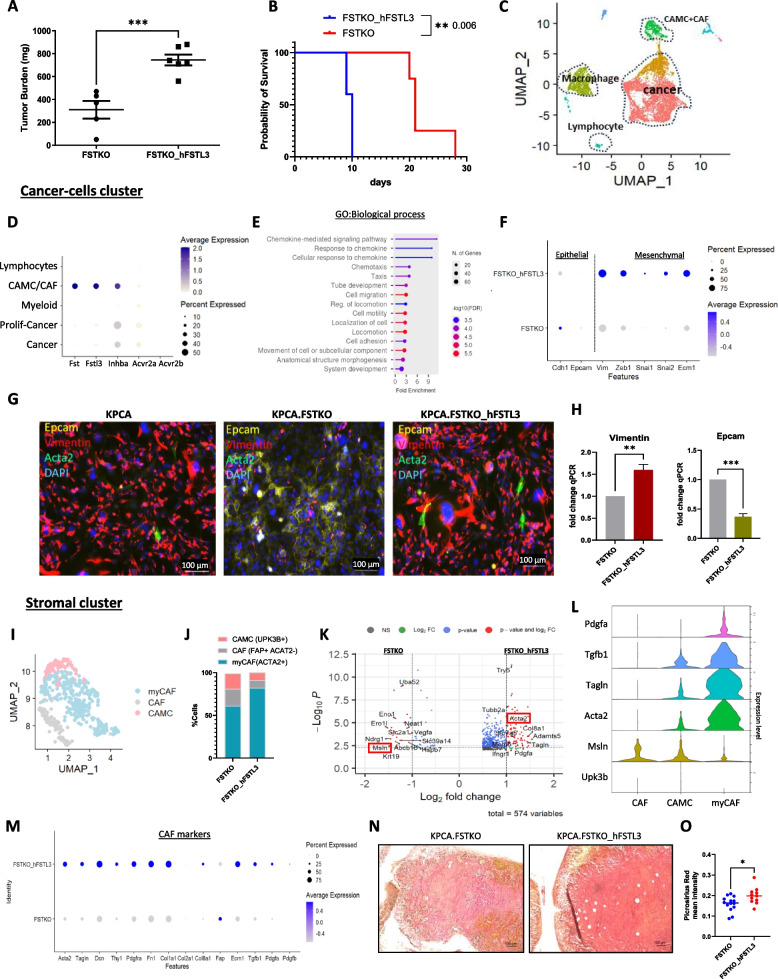
Overexpression of FSTL3 increases tumor development and is associated with a fibrotic microenvironment. **A** Tumor mass of KPCA.FSTKO and KPCA.FSTKO_hFSTL3 model harvested from the peritoneal cavity of mice at endpoint. **B** Kaplan Meier of the survival of KPCA.FSTKO and KPCA.FSTKO_hFSTL3. **C** UMAP of cell populations from the scRNA seq of KPCA.FSTKO and KPCA.FSTKO_hFSTL3. **D** DotPlot representing the transcript expression levels of *Fst*, *Fstl3*, *Inhba*, *Acvr2a*, and *Acvr2b* across different cell identities within the tumor microenvironment of KPCA.FSTKO and KPCA.FSTKO_hFSTL3 models from scRNAseq. Dot size corresponds to the percentage of cells expressing the gene. Dot color intensity indicates average expression level, with darker colors representing higher expression. **E** Pathway analysis (GO biological process) of differential gene expression between KPCA.FSTKO and KPCA.FSTKO_hFSTL3 cancer cell clusters. **F** Dot plot illustrates the expression patterns of key EMT-related genes in cancer cell cluster from KPCA.FSTKO and KPCA.FSTKO_hFSTL3. **G** Representative images show the expression of EPCAM (epithelial marker, yellow), Vimentin (mesenchymal marker, red), ACTA2 (myofibroblast marker, green), and nuclei stained with DAPI (blue) by immunofluorescence. **H** RT-qPCR of *Epcam* and *Vimentin* expression of both KPCA.FSTKO and KPCA.FSTKO_hFSTL3. **I** UMAP of cluster showing the diverse subclusters of stromal compartment including Cancer Associated Mesothelial Cells (CAMC), Cancer Associated Fibroblasts (CAF) and myofibroblasts (myCAF). **J** Proportion (% cells) for each subcluster (CAMC, CAF, myCAF) in KPCA.FSTKO and KPCA.FSTKO_hFSTL3 of the total TME. **K** Volcano Plot of differential gene expression between the KPCA.FSTKO and KPCA.FSTKO_hFSTL3 stromal clusters. **L** VlnPlot showing the expression of secreted factors (TGFB1 & PDGFA) in CAMC (UPK3 K+, MSLN+) and myCAF subtypes (ACTA2+, TAGLN+). **M** DotPlot representing a panel of CAF markers including collagen, extracellular matrix organization and secreted factors transcripts. **N** Image representative tumor section of KPCA.FSTKO and KPCA.FSTKO_hFSTL3 stained with Sirius red. **O** Quantification of Sirius red staining *N*=10)

To investigate the consequences of FSTL3 on tumor growth, we compared KPCA.FSTKO and KPCA.FSTKO_hFSTL3 derived tumors. Following implantation intraperitoneally at 1.10^6 cells/mouse, we observed that FSTL3 overexpression led to a 50% higher tumor mass in mice compared to the KPCA.FSTKO cells at day 10 post-graft (Fig. 4A). Additionally, survival analysis revealed a significant decrease in the survival of mice bearing KPCA.FSTKO_hFSTL3 derived tumors compared to those implanted with KPCA.FSTKO (Fig. 4B).

To investigate the consequence of FSTL3 overexpression on the composition of the TME we conducted an scRNAseq experiment in the KPCA.FSTKO and KPCA.FSTKO_hFSTL3 tumor models (day 10 post-graft) (Fig. 4C). We hypothesized that FSTL3 overexpression in cancer cells not only modifies the cancer cell phenotype, but also modulates immunotherapy response through its action on stromal cell types of the tumor microenvironment. Thanks to the use of a human *hFSTL3* transgene in the KPCA.FSTKO_hFSTL3 model, we were able to discriminate endogenous murine sources of *Fstl3* in the tumor microenvironment. Transcriptomic analysis of KPCA.FSTKO and KPCA.FSTKO_hFSTL3-derived tumor revealed that endogenous mouse *Fst* and *Fstl3* were predominantly expressed by cancer-associated mesothelial and fibroblast cells, which are major components of the stromal tumor microenvironment (Fig. 4D). In contrast, activin A (*Inhba*), one of the main ligands bound by both FST and FSTL3 was primarily secreted by cancer cells, while its type II receptors (*Acvr2a, Acvr2b*) were highly expressed in both cancer cells and fibroblasts (Log2 FC > 0.5) (Fig. 4D).

When we compared the differential gene expression in the cancer clusters from KPCA.FSTKO and hFSTL3-overexpressing tumors, we found a significant enrichment in differentially expressed genes related to pathways in cell migration, adhesion, and locomotion (GO biological process) (Fig. 4E). Underlying these processes, cancer cells overexpressing FSTL3 exhibited increased expression of the genes associated with epithelial-mesenchymal transition (*Vim*, *Zeb-1*, *Snai1/2*) and extracellular matrix organization (*Ecm1*), while expression of epithelial markers (*Epcam*, *Cdh1*) decreased (Fig. 4F) [27, 28]. The differential expression of epithelial (Epcam) and mesenchymal (Vimentin) markers was confirmed by immunofluorescence and qPCR (Fig. 4G/H).

We next evaluated how hFSTL3 overexpression by cancer cells altered the stromal cells in the TME, including cancer-associated mesothelial cells (CAMC) and cancer-associated fibroblasts (CAF). Firstly, among stromal cell types, three cluster subtypes were identified based on marker expression, including CAMC (*Msln+*), CAF (*Fap*+), and MyCAF (myofibroblasts, *Acta2*+) (Fig. 4I). The proportion (percentage of cell) of CAF, MyCAF, and CAMC cells in the stromal cluster was quantified in each model. The KPCA.FSTKO_hFSTL3-derived tumors had a higher proportion of myCAF (82%) and a lower proportion of CAMC cells (9%) than KPCA.FSTKO tumor with 61% myCAF and 18% CAMC in the stromal compartment (Fig. 4J). We next compared the differential gene expression between KPCA.FSTKO and KPCA.FSTKO_hFSTL3 stromal cluster and observed a significantly increased expression (Log2 FC>0.5) of myCAF markers (*Acta2, Tagln*), collagen (*Col8a1)*, and secreted factor (*Pdgfa*) consistent with a myofibroblast phenotype in the KPCA.FSTKO_hFSTL3 tumor. In contrast, in the KPCA.FSTKO tumors, the stromal cluster expressed higher levels of mesothelial markers such as *Krt19* and *Msln*, consistent with a higher proportion of CAMC in this model (Fig. 4K). In addition, we revealed that Tgfb1 was more highly expressed by myCAF compared to CAMC and CAF cells (Fig. 4L).

Since myofibroblast can promote fibrosis and immune evasion [29–33], we next evaluated whether hFSTL3 overexpression accentuated these processes. We compared the expression of collagen, fibronectin, and other extracellular matrix markers that contribute to fibrosis. We found that collagens (*Col1a1*, *Col2a1*, *Col8a1*), fibronectin (*Fn1*), and extracellular matrix component (*Ecm1*) were increased when hFSTL3 was overexpressed in TME (Fig. 4M). Furthermore, the secreted factors *Tgfb1* and *Pdgfa*, which are known to promote fibrosis [34], were also more highly expressed in KPCA.FSTKO_hFSTL3 tumors (Fig. 4M). To evaluate fibrosis, we performed Sirius red staining, which confirmed that the tumors overexpressing FSTL3 exhibited a significantly higher collagen deposition within the tumor microenvironment (Fig. 4N,O), suggesting increased fibrotic areas within these tumors [35].

#### FSTL3 overexpression in TME promotes immune exclusion

**Fig. 5:**
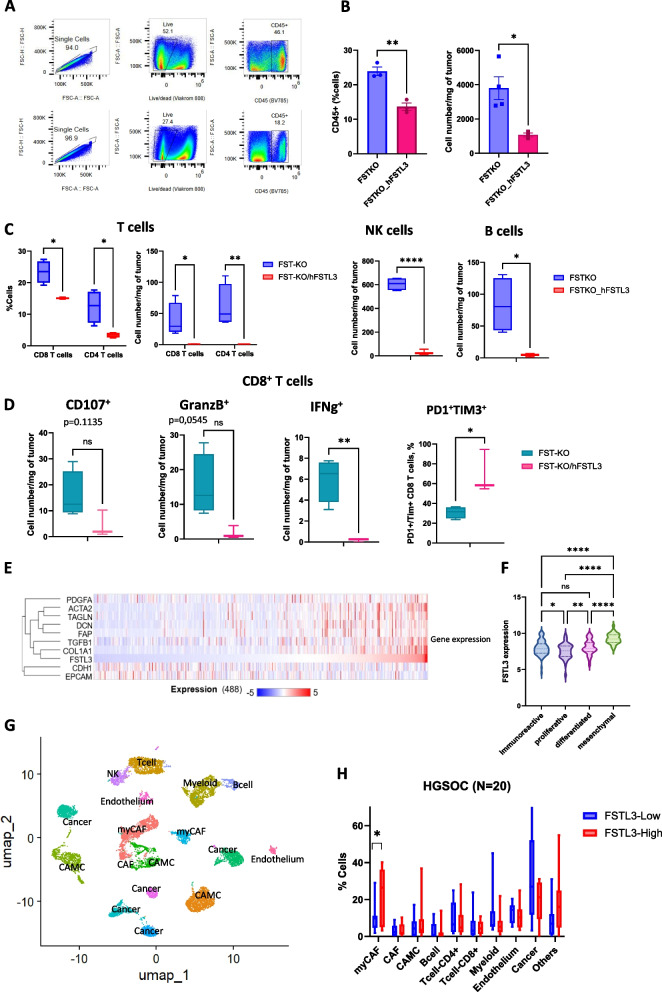
FSTL3 overexpression promotes immunocyte exclusion. **A** Gating strategy to isolate singlet, live, CD45+ cells from KPC.FSTKO and KPCA.FSTKO_hFSTL3 tumors. **B** Percent and number of cells per milligram of tumor of CD45 positive cells in KPCA.FSTKO and KPCA.FSTKO_hFSTL3 tumors (*N*>3). **C** Percent and number of cells of T cells (CD4/CD8), NK and B cells gated on CD45 positive live cells in KPCA.FSTKO and KPCA.FSTKO_hFSTL3 tumors (*N*>3). **D** Expression of CD107, GranzB, IFNg, and ratio of Tim3 on PD1 gated on CD8 T cells population. **E** Heatmap of relative transcript expression of selected genes correlated to FSTL3 expression across patient samples from the TCGA (HGSOC, 486 cases), with red indicating higher expression and blue indicating lower expression. **F** Analysis of TCGA HGSOC tumor transcriptomes (*N*=308) showing expression of FSTL3 by molecular histotype. **G** UMAP of HGSOC of 20 patients from dataset:GSE235931. **H** Proportion of cells present in TME between patients with high and low FSTL3 expression based on median expression (*N*=10 per group)

Given the immunosuppressive role of myCAFs and fibrosis, we investigated whether hFSTL3 overexpression affected the infiltration of immune cells, and particularly CD8+ T cells, given their requirement for PPC treatment response [16]. To this end, we dissociated three tumors per group from treatment-naïve KPCA.FSTKO and KPCA.FSTO_hFSTL3 grafted mice. We stained the cell suspension with specific antibodies, enabling us to quantify immune cell populations by flow cytometry. We measured the total percentage of immune cells (CD45+) and T-cell subsets (CD4+, CD8+) as well as the number of cells per milligram of tumor. We observed a significant reduction in overall immune cell infiltration, with an average decrease of 50% (CD45+) in tumors overexpressing hFSTL3 (Fig. 5A,B). In addition, T cells were markedly reduced in hFSTL3-overexpressing tumors compared to controls with CD4+ (15% versus 25%) and CD8+ (5% versus 15%), suggesting FSTL3-induced immune exclusion may participate in PPC treatment resistance (Fig. 5C). In addition, other immune effector cells, including NK and B cells, were also markedly decreased in FSTL3-expressing tumors (Fig. 5C). To assess the cytotoxic activity of CD8⁺ T cells, we evaluated the expression of CD107a, Granzyme B (GrzB), and IFNγ. These cytotoxic markers were reduced in FSTL3 tumors, while the proportion of TIM3⁺ cells among PD1⁺ CD8⁺ T cells increased, indicating increased T cell exhaustion driven by FSTL3 (Fig. 5D). The myeloid population was also evaluated showing no significant impact in neutrophil infiltration, and a decrease for the macrophages and dendritic cells (Fig. S1A). The gating strategies to identify the cell populations are represented in Fig. S1B.

To determine if hFSTL3 overexpression was also associated with a more fibrotic and immunosuppressive TME in human HGSOC tumors, we analysed data from The Cancer Genome Atlas (TCGA) dataset, containing bulk RNAseq from 486 HGSOC cases [36]. We found an association between high FSTL3 transcript levels and expression of CAF markers and fibrosis markers (COL1 A1, TGFB1, FAP, DCN, TAGLN, ACTA2, PDGFA), previously identified as upregulated in KPCA.FSTKO_hFSTL3 mouse tumors (Fig. 5E). In contrast, epithelial markers (EPCAM, CDH1) showed an inverse correlation with FSTL3. We next sought to evaluate if FSTL3 overexpression was associated with a particular ovarian cancer molecular histotype [7] (Fig. 5E). To do so we compared FSTL3 transcript levels from an RNAseq dataset of the TCGA ovarian cancer UNC hub (*n*=308) annotated with the molecular subtypes as proliferative, immunoreactive, differentiated, and mesenchymal, and found that FSTL3 was significantly higher in the mesenchymal subtype compared to all others (Fig. 5F). These data suggest that FSTL3 overexpression also promotes a more mesenchymal phenotype in human tumors.

To further confirm the role of FSTL3 in inducing immune evasion in human patients, we analyzed the TME using single-cell RNA-seq data from twenty HGSOC patients (Fig. 5G). Patients were stratified into two groups based on median FSTL3 expression levels: FSTL3-high (*N*=10) and FSTL3-low (*N*=10). We then compared the cellular composition of the TMEs between the two groups based on % marker expression. Similar to observations in the mouse TME, FSTL3-high human samples exhibited a significant increased proportion of myCAF cells and a trend for higher content in other stromal cells (CAMC, CAF), correlating with a marked reduction of CD4+ and CD8+ T cells. These findings suggest that FSTL3 overexpression may promote a high abundance of CAFs and contribute to T cell exclusion in human tumors (Fig. 5H).

## Discussion

Ovarian cancer, and particularly HGSOC, continues to pose a significant clinical challenge due to its frequent late-stage diagnosis and high recurrence rates. The identification of FST as a potential player in immune evasion and treatment resistance has paved the way for exploring the role of other follistatin homologs such as FSTL3 in ovarian cancer [16].

In this study, we identified significant overexpression of both FST and FSTL3 in 96 ascites samples from ovarian cancer patients. We found that FSTL3 levels were generally higher than FST, both in the ascites and in the tumors of HGSOC patients. Moreover, elevated FSTL3 protein levels in ascites of patients with BRCA1 wildtype tumors, where treatment options are notably limited, were significantly associated with poorer survival outcomes. This correlation between overexpression of FSTL3 and poor outcome may not be unique to ascites nor ovarian cancer as it was also observed at the transcriptional level across multiple solid tumor types including pancreatic, lung, and colorectal cancers through analysis of data from the TCGA. Importantly, we found that FSTL3 could also be detected in the serum of mice bearing ovarian tumors, and observed a strong relationship between FSTL3 concentration and tumor growth. We observed marked decreases in serum FSTL3 associated with treatment responses in mice, and in a patient receiving neoadjuvant chemotherapy through serial sampling of ascites. While cancer cells express FST/FSTL3, our scRNAseq and spatial transcriptomic analyses suggest that tumor stromal cells may be the predominant source of this factor in the TME. Importantly, FSTL3 background levels in the blood of nulliparous female mice were low, likely due to the modest expression of FSTL3 in tissues other than the placenta [37, 38], suggesting it may represent a sensitive clinical biomarker to track HGSOC progression and treatment response.

In a previous study we showed that knocking out FST in the “KPCA” model of CCNE1-amplified HGSOC promoted sensitivity to a combination immunotherapy treatment (PPC: Prexasertib, PD-1, and CTLA-4 blockade) [16].

In this study, we show that overexpression of human FSTL3 in this model (KPCA.FSTKO_hFSTL3) restores complete resistance to PPC, highlighting the redundant function of FST and FSTL3 in mediating immunotherapy resistance. The effectiveness of PPC therapy in the KPCA.FSTKO model was found to result from a synergistic effect between Prexasertib and immune checkpoint inhibitors (anti-PD1 and anti-CTLA4). Prexasertib, a checkpoint kinase 1 (CHK1) inhibitor, has shown potential for cancers that exhibit high DNA replication stress and impaired DNA damage response pathways [37–39], such as HGSOC. A phase II trial evaluating Prexasertib in BRCA wild-type, platinum-resistant HGSOC showed some tumor shrinkage but limited overall responses [40, 41]. Other drugs acting on the same DNA damage response pathway, such as ATR inhibitors, have shown more promise in ovarian cancer [42], and are being investigated as potentiators of immune checkpoint blockade [43–46], which are otherwise often ineffective on their own [47]. Thus, combinations of immune checkpoint inhibitors with targeted therapies, and a better understanding of the mechanism of immunotherapy resistance, could provide new avenues for curative treatment in ovarian cancer.

We hypothesized that FSTL3 overexpression contributes to immunotherapy resistance by altering the tumor microenvironment: promoting a more mesenchymal tumor phenotype, increasing fibrosis by promoting myCAF development, suppressing immunocyte infiltration, and promoting T cell exhaustion. These changes may be in direct response to FSTL3 neutralizing canonical targets such as activin A, which would otherwise attract and modulate immunocytes, for example by potentiating CD8+ T cells [48], or indirectly through modulating fibroblast growth, migration, and fibrosis via the secretion of other factors such as PDGF (Platelet-Derived Growth Factor) and TGF-β1 (Transforming Growth Factor Beta 1) [49, 50], contributing to immunocyte exclusion. Aberrant PDGF signaling has been implicated in the activation of stromal cells, such as fibroblasts, which promote an immunosuppressive niche [51]. Similarly, Elevated TGF-β1 levels in the tumor microenvironment drive epithelial-to-mesenchymal transition (EMT), immune evasion, and extracellular matrix remodeling, processes that are critical for tumor invasion and metastasis [52]. Moreover, TGF-β1 is a potent immunosuppressive cytokine that inhibits T-cell function and supports regulatory T-cell differentiation, thereby undermining anti-tumor immunity [53, 54]. CAFs and other stromal cells, such as CAMCs, promote immune evasion [55–57] and may limit the effectiveness of immunotherapy treatments in solid tumors [28, 58]. Fibrotic processes are common in cancers such as pancreatic, breast, lung, and liver cancer, where extensive fibrosis is frequently linked to poor prognosis [59] and can reduce immunocyte infiltration [32, 34, 60]. In our mouse model, FSTL3 overexpression led to fibrosis, as indicated by significantly increased collagen deposition, which in turn could have impeded immune cell infiltration.

In our syngeneic ovarian cancer model, FSTL3 overexpression significantly altered the tumor immunocyte composition, by reducing total CD45+ infiltration, and specifically NK cells, B cells, T cells, macrophages, and dendritic cells. Notably, the populations of CD4+ and CD8+ T cells were markedly reduced, with the latter adopting an exhausted phenotype with decreased expression of IFNg [61], and increased TIM3/PD1 [62–64]. A similar immune profile was also observed in lung adenocarcinoma, where high FSTL3 levels correlated with a reduction of immune cell infiltration, including T lymphocytes and B lymphocytes in the TME [65], or in gastric and colorectal cancer where it was associated with poor prognosis, EMT, fibrosis, and changes in immunocyte composition. However, the precise mechanism leading to these changes, and particularly the ligands modulated by FSTL3 in the context of the TME that directly regulate immunocytes, remain to be identified in ovarian cancer [66–70]. The T cell exhaustion promoted by FSTL3 overexpression likely contributed to the observed resistance PPC therapy in our syngeneic mouse model. Indeed, expression of PD1 and TIM3 in tumor-infiltrating lymphocytes is associated with immune checkpoint resistance, irrespective of PD-L1 expression in tumors such non-small cell lung cancer [71]. Furthermore, our observation that FSTL3 overexpression drives EMT in cancer cells autonomously in the KPCA cell line, and that its expression is higher in HGSOC tumors of the mesenchymal histotype in the TCGA, suggest that FSTL3-mediated cancer cell EMT could indirectly contribute to the observed immune checkpoint resistance, through their interactions with T cells [72].

In summary, our data suggest that ovarian tumors secrete FSTL3 in the blood and ascites and that it is an indicator of poor prognosis. Experimental overexpression of FSTL3 in murine ovarian cancer cells changed the TME by promoting cancer cell EMT, increasing stromal fibrosis, reducing immunocyte recruitment, and promoting T cell exhaustion, which we speculate contributed to the induced resistance to PPC combination immunotherapy. Thus, FSTL3 may represent both a new secreted biomarker to optimize patient stratification, monitor disease burden and treatment response, and a potential target to overcome resistance to immune checkpoint therapies in ovarian cancer. Further research is needed to build upon these findings and develop strategies to neutralize FSTL3 to meet the urgent demand for more effective treatments in HGSOC.

## Materials and methods

### Human samples

Ascites samples from patients with various subtypes of ovarian cancers (96 samples) along with sections of high-grade serous ovarian cancer tumors were generously donated by female patients to the Massachusetts General Hospital, in accordance with an IRB-approved protocol (2007P001918).

Spatial transcriptomics analysis from tumor sections were donated by the Biological Resources Center of Montpellier Cancer Institute (ICM), France, and were collected following the French regulations under the supervision of an investigator. The collection was declared to the French Ministry of Higher Education and Research (declaration number DC-2008–695).

### ELISA

ELISA assays for human samples were performed using ANSH lab FST and FSTL3 kits. For mouse serum and ascites samples, RayBio mFST and AVIVA mFSTL3 kits were employed. ELISAs were conducted according to the manufacturer’s protocols. Optimal dilutions were determined before processing all samples. Absorbance was measured at 450 nm using a microplate reader (Pherastar), and protein concentrations were quantified based on standard curves generated following the kit instructions.

### Cell Lines and 2D cell culture

Ovarian cancer cell lines KPCA, KPCA.FSTKO, KPCA.FSTKO_hFSTL3 were maintained in Dulbecco’s modified Eagle’s medium tissue culture medium supplemented with 5% fetal bovine serum (FBS) and 1% penicillin-streptomycin. All cells were cultured at 37 °C in 5% CO2.

To generate the KPCA.FSTKO_hFSTL3 cell line, we used the previously described KPCA.FSTKO cell line [16], and transfected it with a pcDNA3.1 expression plasmid for hFSTL3 (Genscript). A stable clone was selected under geneticin selection based on FSTL3 secretion in conditioned media as measured by ELISA. The SBE-luciferase reporter cells were a gift of Tom Thompson.

### RNA isolation and quantitative real-time PCR (RT-qPCR) analysis

Total RNA was isolated from cancer cells or tumors using the Qiagen RNA extraction kit. The cDNA synthesis was carried out with the SuperScript III First-Strand Synthesis System for RT-PCR (Invitrogen). The cDNA was then combined with primers and iQ SYBR Green Supermix (#1708882, Bio-Rad) in a 96-well plate. Reverse transcription was performed on a T100 Thermal Cycler (Bio-Rad), and qPCR was conducted using the CFX96 Touch Real-Time PCR Detection System (Bio-Rad). Gene expression levels were determined relative to housekeeping genes (GAPDH or HPRT) pre-designed from IDT biotech©, with expression quantification based on cycle threshold (Ct) values transformed using the 2^(-ΔCt) method, and the results were analyzed and plotted with GraphPad PRISM.

### Single-cell RNA sequencing

Single-cell RNA sequencing was performed using a 10X Genomics 3’ kit following manufacturer’s instructions. We dissociated tumors derived from KPCA.FSTKO and KPCA.FSTKO_hFSTL3 grafted mice (*N* = 4 pooled tumors per genotype) as previously described [55]. We targeted 10,000 cells per sample, and obtained a total final cell count of 7582 for KPCA.FSKO and 4244 for KPCA.FSTKO_hFSTL3 after data analysis and quality control. The data analysis was performed using Cell Ranger (version 3.1.0) with standard parameters. Samples were aligned against the refdata-gex-mm10-2020-A reference sequences and were analyzed using Seurat library (R version 4.3.3). Standard pre-processing workflow was applied independently to each sample. Samples were filtered for mitochondrial percentage <20% and unique feature counts over 200 and less than 8000. Samples were normalized then merged using ‘‘merge’’ function in Seurat. Standard parameters for visualization and clustering were performed throughout the analysis. Markers for each level of cluster were identified using FindAllMarkers in Seurat. The 10X scRNAseq datasets are available on the Gene Expression Omnibus (GEO) platform under accession number GEO: GSE283618.

### Spatial transcriptomic

The spatial transcriptomic analysis was conducted using the Xenium platform (10x Genomics) on three treatment-naive high-grade serous ovarian cancer tumor samples. The expression levels of FST and FSTL3 transcripts were quantified across 12 representative regions per tumor to capture the spatial heterogeneity. The data was processed and analyzed using Xenium software (10x Genomics), enabling precise transcript quantification and spatial mapping of gene expression patterns. The colocalization was quantified using the Coloc2 plugin in ImageJ and corresponds to the percentage of area overlap between FST or FSTL3 and either stromal or Epcam markers.

### Single-cell analysis of patient datasets

FSTL3 transcript expression data were extracted from the GSE235931 dataset of 14 high-grade serous ovarian cancer tumors and analyzed using RStudio with Seurat. Patients were categorized into two groups based on their FSTL3 expression levels: FSTL3-High patients had an average expression above the population median (≥0.08), while FSTL3-Low patients had expression levels below the population median (<0.08). The tumor microenvironments of the FSTL3-High and FSTL3-Low groups were compared using specific markers indicative of cell phenotypes of interest, including ACTA2, FAP, UPK3B, CD4, CD8, EPCAM, MS4 A1, and CD14. To further analyze the differences between these groups, the percentage of cells expressing these specific markers was calculated using the WhichCells command from the Seurat package.

### TCGA transcriptomic analysis

Survival analysis based on FSTL3 and FST expression was conducted using data extracted from The Cancer Genome Atlas (TCGA) from 349 ovarian cancer patients. Additionally, FSTL3 expression was analyzed in relation to survival outcomes in 497 lung cancer patients, 176 pancreatic cancer patients, and 307 colorectal cancer patients (Ov-TCGA, LUSC-TCGA, PAAD-TCGA, COAD-TCGA datasets) [73, 74]. Patients were categorized into FST/FSTL3-High or FST/FSTL3-Low groups based on their expression levels relative to the median expression across all patients. Specifically, FST/FSTL3-High was defined as expression above the median, while FST/FSTL3-Low was defined as expression below the median. Statistical analyses were performed using GraphPad Prism, with survival curves compared using the Mantel-Cox log-rank test.

To evaluate FSTL3 expression in HGSOC by molecular histotype, we extracted FSTL3 transcript levels from an RNAseq dataset of the TCGA ovarian cancer (OV) UNC hub subset (*n*=308) with metadata identifying gene_expression_subtypes as proliferative, immunoreactive, differentiated, and mesenchymal. This level 3 data was downloaded from the UCSC Xena repository [75]. The transcript numbers from Illumina HiSeqv2 data were log-transformed (log2(norm_count+1).

### Sirius red staining

Paraffin-embedded tissue sections were deparaffinized and rehydrated through graded alcohols to water. The sections were stained in a picro-Sirius Red solution, prepared by dissolving 0.5 g of Sirius Red (Sigma Aldrich, #365548) in 500 mL of a saturated aqueous solution of picric acid, for one hour. After staining, the sections were washed in two changes of 0.1M glacial acetic acid. Excess water was removed from the slides by blotting with damp filter paper. The slides were then dehydrated in three changes of absolute ethanol, cleared in xylene, and mounted in a resinous medium (VectorLabs). Picrosirius Red intensity was analyzed using the CellProfiler Software. Briefly, following image acquisition by light microscopy, the red channel marking collagen and yellow channels were split and the yellow was subtracted from the red one. The “smooth” module was then used before identifying the area covered by the stain. The mean intensity of this delimitated area was then quantified.

### Flow cytometry analysis

After dissociation of KPCA.FSTKO (N=4) and KPCA.FSTKO_hFSTL3 tumors (N=4), 5×10^4 cells were added in each well of a 96-well V-bottom plate. Cells were then immunostained with antibodies according to the manufacturer’s recommendations, washed 3 times and fixed with 1% Paraformaldehyde. Live and dead reagent (Thermofisher, L10119) was used to discriminate the live cells and antibody panels were used for T cells and myeloids. Immunostained cells were run on Cytek Aurora Flow cytometer and analyses were performed with FlowJo software.

### Immunofluorescence

Tumors were fixed overnight in 10% neutral buffered formalin. The fixed samples were subsequently processed through an alcohol series, cleared with xylene, and embedded in paraffin. Sections were cut to a thickness of 10 µm and mounted onto glass slides. Dewaxing was performed by incubating at 60 °C for 60 minutes, followed by rehydration through a series of graded ethanol washes. Antigen retrieval was achieved using citrate buffer in a pressure cooker. Slides were then incubated for 1 hour at room temperature in blocking buffer, followed by three washes with PBS. The primary antibodies (anti-Epcam, anti-Vimentin, anti-Acta2) used at the concentration recommended by the manufacturer (Thermofisher), were applied overnight at 4 °C. After washing, the slides were incubated for 1 hour with a fluorophore-conjugated secondary antibody (anti-Mouse-488nm/anti-rabbit-555nm, Thermofisher). To visualize the stained proteins, sections were counterstained with DAPI for 5 minutes and then washed with PBS. Coverslips were mounted using Vectashield mounting medium to prevent fluorescence quenching. Fluorescent signals were visualized and analyzed using an epifluorescence microscope.

### Animal experiments

This study was performed according to experimental protocols 2009 N000033 and 2019 N000068, approved by the Massachusetts General Hospital Institutional Animal Care and Use Committee. All experiments were made with 12–16 weeks female C57BL/6 J mice purchased from the Jackson Laboratory. In each experiment, we used 4 to 5 mice per group.

#### Cancer cell graft

In all in vivo experiments, 1.10^6^ cells of KPCA, KPCA.FSTKO, or KPCA.FSTKO_hFSTL3 were injected intraperitoneally.

Blood samples were collected using a 5 mm lancet on the submandibular vein, alternating the collection site between cheeks. A one-week healing interval was observed between procedures, while ensuring compliance with the maximum blood volume limits. Upon euthanasia, cardiac blood collection was performed. Serum was separated by allowing the collected blood (submandibular vein or cardiac collection) to clot at room temperature for 30 minutes, followed by centrifugation at 2,000 x g for 10 minutes at 4 °C. The supernatant (serum) was carefully collected, labeled and stored at −20°C.

Treatments began on day 5 post-graft and were administered intraperitoneally twice a week with the indicated doses during 4 weeks for KPCA.FSTKO and 2 weeks for KPCA.FSTKO_hFSTL3. For immunotherapy, anti-CTLA4 (50 μg/mouse; Bio X Cell, BE0164) and anti-PD1 (50 μg/mouse; Bio X Cell, BP0273) antibodies were used. Prexasertib (Selleckchem, S7178; 10 mg/kg) was administered either as a monotherapy or in combination with the immunotherapies and was resuspended according to the manufacturer’s instructions. Control mice were injected with 10% DMSO (vehicle) and an isotype control antibody (InVivoMAb mouse IgG2a isotype control, unknown specificity & InVivoPlus rat IgG2a isotype control, anti-trinitrophenol).

Bilateral oophorectomy was performed under Isoflurane anesthesia. Aseptic precautions were followed, and all surgical instruments were autoclaved prior to the procedure. The animal preparation included shaving the fur around the incision site, placing the animal on a warming pad, applying surgical soap scrub three times, and draping the animal. Eye lubricant was applied to prevent dryness. Carprofen (2–5 mg/kg) was administered subcutaneously just before surgery and continued every 12–24 hours for up to 72 hours postoperatively. Small incisions were made in the dorsolumbar region through the skin and body wall. The ovaries were exteriorized, followed by section and hemostasis using a cauterizer. The fascia was closed with sutures, and the skin was closed with surgical clips. Triple Antibiotic ointment (Bacitracin 400 U, Neomycin 3.5 mg, Polymyxin B 5,000 U.) was applied once. Mice were closely monitored on a warming mat post-surgery and then transferred to a cage with warm bedding. They were continuously monitored until they were fully ambulatory. Staples were removed at day 7 under isoflurane anesthesia.

#### Monitoring

Mice were monitored regularly for health and survival according to an IACUC approved protocol, euthanasia was performed if animals exhibited signs of poor body condition or distress.

### Statistical analysis

Statistical analysis was performed using GraphPad Prism 10.1.1 software. An independent sample t-test was used unless otherwise specified. Paired t-test was used to compare baseline and endpoint FST or FSTL3 levels by mouse. For comparisons involving more than two groups, one-way ANOVA was employed, or Kruskal-Wallis was used when data distribution was non-Gaussian. A *p*-value of ≤ 0.05 was considered statistically significant. Data are represented as the mean ± standard error of the mean (SEM).

## Supplementary Information

Supplementary Material 1: Figure S1. FSTL3 overexpression promotes immunocyte exclusion. A) Gating strategy to identify myeloid and lymphocytes population in KPCA.FSTKO and KPCA.FSTKO_hFSTL3 tumors by flow cytometry. B) Number of neutrophils, macrophages and dendritic cells per milligram of tumor in KPCA.FSTKO and KPCA.FSTKO_hFSTL3 tumors (*N*>3).

Supplementary Material 2: Table S1. Concentration of FST and FSTL3 in Ascites samples from patients and their clinical characteristics.

## Data Availability

The 10X scRNAseq datasets generated in this study are available on the Gene Expression Omnibus (GEO) platform under accession number GEO: GSE283618
