# Supplementary figures and images for: FSTL3 is a biomarker of poor prognosis and associated with immunotherapy resistance in ovarian cancer
Source: J Exp Clin Cancer Res. 2025 Sep 30;44:271. doi: 10.1186/s13046-025-03425-4 (PMC12487040; doi:10.1186/s13046-025-03425-4)

## A Myeloid cells

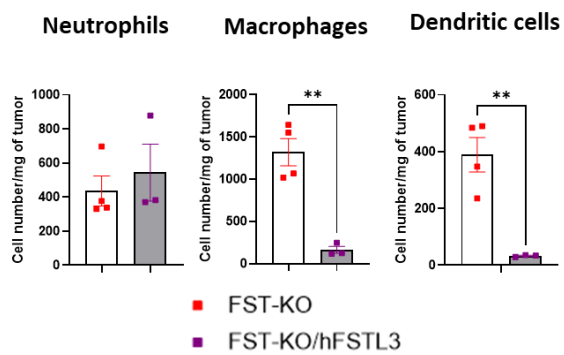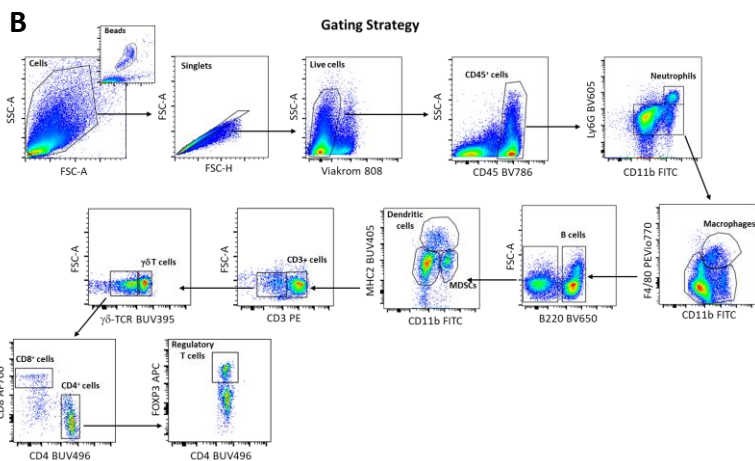

Supplement: Supplementary file 1 — Supplementary Material 1: Figure S1. FSTL3 overexpression promotes immunocyte exclusion. A) Gating strategy to identify myeloid and lymphocytes population in KPCA.FSTKO and KPCA.FSTKO_hFSTL3 tumors by flow cytometry. B) Number of neutrophils, macrophages and dendritic cells per milligram of tumor in KPCA.FSTKO and KPCA.FSTKO_hFSTL3 tumors (N>3). [file 13046_2025_3425_MOESM1_ESM.pdf]
